# The Role of Rare Coding Variants in Parkinson's Disease GWAS Loci

**DOI:** 10.3389/fneur.2019.01284

**Published:** 2019-12-13

**Authors:** Elisabeth Luisa Germer, Sophie Imhoff, Carles Vilariño-Güell, Meike Kasten, Philip Seibler, Norbert Brüggemann, International Parkinson’s Disease Genomics Consortium, Christine Klein, Joanne Trinh

**Affiliations:** ^1^Institute of Neurogenetics, University of Lübeck, Lübeck, Germany; ^2^Department of Medical Genetics, Centre for Applied Neurogenetics, University of British Columbia, Vancouver, BC, Canada

**Keywords:** Parkinson's disease, GWAS, rare variants association analyses, exome analysis, STAB1, SH3GL2, NOD2

## Abstract

**Introduction:** Genome-wide association studies (GWAS) have identified multiple loci associated with Parkinson's disease (PD) risk. The presence of rare variants within these loci that may account for the increased susceptibility requires further investigation.

**Methods:** Using exome sequencing, we performed a comprehensive rare variant screen of genes located within 56 novel PD loci. We first analyzed exomes from 109 subjects in the discovery cohort (85 diagnosed with PD and 24 healthy controls) and filtered for rare coding variants with minor allele frequency <0.01 and combined annotation-dependent depletion > 15. Further investigation of exome data from a replication cohort of 2,859 European patients with PD (International Parkinson's Disease Genomics Consortium) and 24,146 non-Finnish European controls from gnomAD were used for association testing of specific rare variants found in the discovery cohort.

**Results:** Our genetic screening identified 54 potential disease-relevant variants in 71 genes in 109 subjects. Six out of 54 variants were found in two or more patients and were not observed in healthy controls: *DNAH1* p.A3639T, *STAB1* p.S1089G, *ANK2* p.V3634D, *ANK2* p.R3906W, *SH3GL2* p.G276V, and *NOD2* p.G908R. Replication in the International Parkinson's Disease Genomics Consortium (IPDGC) confirmed the association with PD risk for three out of the six identified variants (*STAB1* p.S1089G, *SH3GL2* p.G276V, and *NOD2* p.G908R) (*p* < 10^−3^).

**Conclusion:** Our study suggests that some of the associations identified in PD risk loci can be ascribed to rare variants with likely functional effects that modify PD risk.

## Introduction

Parkinson's disease (PD) is the second most common neurodegenerative disease. To date, 5–10% of PD is explained by monogenic causes (1, 2). However, a large part of PD still remains genetically unexplained, although heritability estimates show that the genetic components account for ~27% (3, 4). Thus, a substantial proportion of genetic influence on PD remains to be elucidated. Genome-wide association studies (GWAS) in PD patients have identified genetic loci, which typically nominate regions of association but do not pinpoint causal variants. Both common and rare coding variants in *SNCA* and *LRRK2* have been reported to be associated with PD. *SNCA* p.A53T was the first identified pathogenic mutation for PD (5), and subsequent GWAS nominated common risk variants for PD in *SNCA* (6). Similarly, *LRRK2* harbors the most common pathogenic mutation in familial PD, p.G2019S, accounting for up to 40% of Tunisian Arab-Berbers and 20% in Ashkenazi Jews with familial PD (7, 8). An association in the *LRRK2* locus was identified in a recent GWAS (4). This supports the hypothesis that genes identified in GWAS can harbor biologically relevant rare coding variants and account for the observed association. Two recent GWAS (9, 10) identified 56 novel risk loci for PD. As an increasing number of risk loci are identified through GWAS meta-analyses, it is important to further investigate whether associated genes harbor rare variants that contribute to risk. In this study, we performed an exploratory analysis on the impact of rare coding variants within novel GWAS loci and its relationship with PD risk.

## Methods

### Patient Demographics

We studied exomes from 109 subjects who are from Germany, recruited in Lübeck (85 with PD and 24 healthy controls); 23 patients were known to carry either a pathogenic or likely pathogenic variant in genes described in PD (11) (i.e., *PRKN, SNCA, PINK1, PLA2G6, GBA, LRRK2*) (Supplementary Table 1). A previously published analysis had identified patients with likely pathogenic or pathogenic genetic variants (11). We included these 23 patients with a known genetic cause to assess the presence of double mutations, albeit on an exploratory level as there is no sufficient power to identify disease modifiers. Exome sequencing was performed on all 109 individuals, and exome variants located within the 56 GWAS PD loci were selected for further analysis (9, 10). In our exome cohort, 17% of patients had a positive family history (FH), 51% had negative FH, and 32% had no FH available. Positive FH is defined as having any family member affected with PD. Local ethics approval was obtained from the Research Ethics Board of the University of Lübeck. All patients were examined and diagnosed by movement disorder specialists (MK, NB, and CK). All participants provided informed consent before donating a blood sample for genetic analysis and are from Germany or of other European descent.

### Exome Sequencing

Exome sequencing was performed with Illumina's Nextera Rapid Capture Exome Kit followed by massively parallel sequencing on a NextSeq500 Sequencer (Illumina, San Diego, CA, USA). Raw sequencing reads were converted to fastq format using bcl2fastq software (Illumina). Using an in-house developed pipeline for exome data analysis, the reads were aligned to the human reference genome (GRCh37, hg19 build) with Burrows-Wheeler algorithm software and the mem algorithm. Alignments were converted to binary bam file, and variant calling was performed using three different variant callers (GATK HaplotypeCaller, freebayes, and samtools). Variants were annotated using Annovar and in-house *ad hoc* bioinformatic tools (12).

### GWAS Loci Screening: Variant Selection

**Figure 1:**
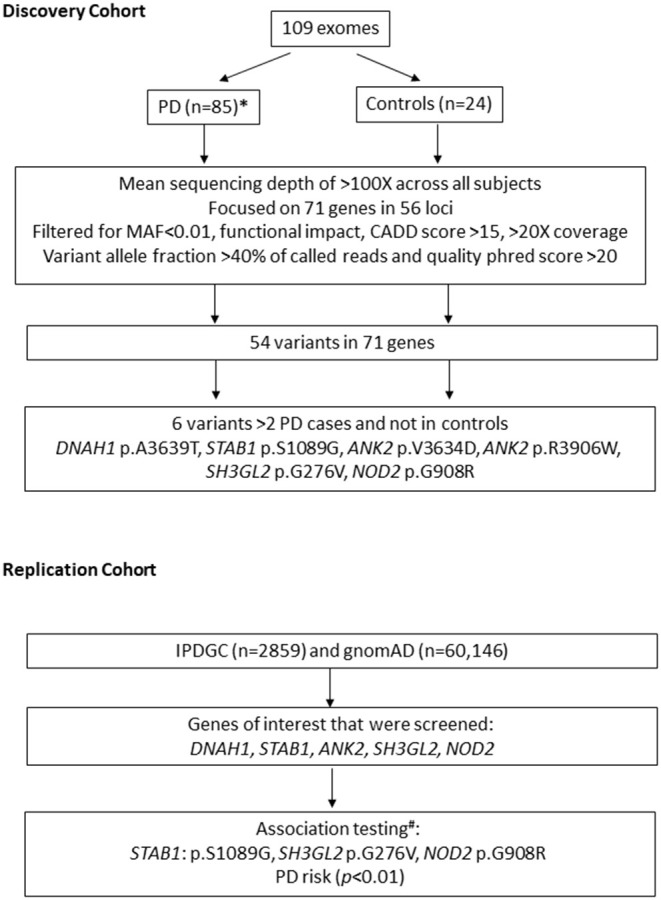
Overview of study design. *n*, number of subjects; MAF, minor allele frequency; IPDGC, International Parkinson's Disease Genetics Consortium; gnomAD, Genome aggregation database; PD, Parkinson's disease. * 23/85 patients have likely pathogenic variants in known genes for PD. # performed using non-Finish European controls *n* = 24,146.

We investigated 71 susceptibility genes within 56 loci identified in the two most recent PD GWAS (9, 10), which nominated 71 susceptibility genes within 56 loci (Supplementary Table 2). The two most recent GWAS publications were used: 32 possible genes within 17 identified associated loci were nominated by Chang et al. (9), and 39 possible genes within 39 identified associated loci were nominated by Nalls et al. (4). We first examined our discovery cohort (109 exomes) to detect novel disease-associated variants for PD in the 71 nominated genes, although power was a limitation. We selected variants with (1) minor allele frequency <0.01 in Genome Aggregation Database v2.1.1(gnomAD) (13), (2) functional impact: non-synonymous, stop-gain, frameshift, and splicing variants, (3) combined annotation-dependent depletion (CADD) score > 15, and (4) Phred quality score > 20, coverage > 20×, and variant allele fraction > 40% of called reads. All the selected variants identified in two or more patients were validated by Sanger sequencing. An overview of the study design is illustrated in Figure 1.

### Replication Association Test Statistics

Subsequent association tests were performed in the International Parkinson's Disease Genomics Consortium (IPDGC) replication cohort for six nominated variants in five genes (Figure 1, Table 1) (*DNAH1, STAB1, ANK2, SH3GL2*, and *NOD2*). Summary statistics were obtained from the exome sequencing data on a PD database query. Each patient carrying variant of interest from these five genes was obtained from the full IPDGC cohort for further association testing. Genetic association testing on specific variants found in the original German discovery cohort was also performed by comparing the 2,859 unrelated individuals from the IPDGC and 24,146 individuals from the non-Finnish European population (we excluded the Finnish European population because of reduced genetic diversity) from gnomAD (13) using Fisher's exact test. After correcting for multiple testing, *p* values < 0.0085 were considered statistically significant.

**Table 1:** Patients with candidate variants in nominated GWAS loci.

**Mutation**	**ID**	**Sex**	**Age**	**AAO**	**FH**	**Population**	**Pathogenic variants**
**(A) IDIOPATHIC PD**							
*DNAH1 p.A3639T*	L-1523	Female	65	32	No	Polish	n.a
*ANK2 p.V3634D*	L-7378	Male	65	56	No	German	n.a
	L-1371	Female	58	32	No	German	n.a
	L-2035	Male	35	30	n.a.	Polish	n.a
*ANK2 p.R3906W*	L-377	Male	n.a.	28	Yes	German	n.a
	L-3672	Male	64	28	No	G‘erman	n.a
*SH3GL2 p.G276V*	L-7888	Male	82	80	No	German	n.a
	L-3094	Female	61	51	n.a	German	n.a
*NOD2 p.G908R*	L-587	Female	44	28	No	German	n.a.
**(B) CARRIERS OF PATHOGENIC VARIANTS**							
*DNAH1 p.A3639T*	L-295	Female	n.a.	45	Yes.	German	*LRRK2* het p.R1441C
	L-2501	Male	n.a.	n.a.	Yes	German	*LRRK2* het p.R1441C
*STAB1 p.S1089G*	L-2124	Female	68	53	Yes	German	*PINK1* homo p.Gln456*
	L-2126	Female	72	47	Yes	German	*PINK1* homo p.Gln456*
*ANK2 p.V3634D*	L-3035	Male	40	35	No	n.a.	*PRKN* het Ex3-4 Del.+ het Ex7-9 Dupl
*SH3GL2 p.G276V*	L-649	Male	n.a.	16	Yes	German	*PRKN* het p.R275W
*NOD2 p.G908R*	L-1888	Female	25	19	No	German	*PRKN* het p.R275W and Ex1 Dupl

*ID, identifier; AAO, age at onset; FH, family history; n.a., not available; het, heterozygous; homo, homozygous; Dupl, duplication*.

## Results

### Identification of Rare Variants in PD Loci and Candidate Genes

Exome sequencing on 109 individuals resulted in an overall average coverage >100×. The average PD onset age is 35.9 years (range, 6–80) across 85 patients. The average age at collection is 55.8 years (range, 19–89) for PD patients and 66.4 years (range, 56–81) across 24 controls. The percentage of male patients is 62% in the patient cohort and 38% in the control cohort. We did not have the power for association analysis when separating individuals with a FH and sporadic cases. No individuals were related to each other besides the one set of siblings reported in this study. After applying our standard filtering criteria of (1) minor allele frequency < 0.01 in Genome Aggregation Database (gnomAD), (2) functional impact: non-synonymous, stop-gain, frameshift, and splicing variants, (3) CADD score > 15, and (4) Phred quality score > 20, coverage > 20×, and variant allele fraction > 40% of called reads, we identified 54 unique variants in 71 candidate genes; of these variants, 49 were not observed in healthy controls (Supplementary Table 3). However, we acknowledge the limitation of the small sample size of our discovery cohort. Furthermore, 6 out of 49 variants were present in multiple PD cases and located in five genes (*DNAH1*: c.10915G>A: p.A3639T (NM_015512), *STAB1*: c.3265A>G: p.S1089G (NM_015136), *ANK2*: c.10901T>A: p.V3634D (NM_001148), *ANK2*: c.11716C>T: p.R3906W (NM_001148), *SH3GL2*: c.827G>T: p.G276V (NM_003026), *NOD2*: c.2722G>C: p.G908R (NM_022162) (Table 1).

### Rare Variants in Idiopathic PD

Out of 62 patients with idiopathic PD, we found 9 rare variants fulfilling the selection criteria and not in controls. Three patients (L-7378, L-1371, and L-2035) are carriers of an *ANK2*: p.V3634D variant. Two patients are *ANK2* p.R3906W carriers with an age at onset (AAO) of 28 years (L-377 and L-3672). Two patients are carriers of the *SH3GL2* p.G276V variant (L-3094 and L-7888). L-3094 and L-7888 have an AAO of 51 and 80 years, respectively. *NOD2* p.G908R was found in two patients with a negative FH of PD. The other *NOD2* p.G908R carrier (L-587) had an AAO of 28 years.

### Rare Variants in Pathogenic Variant Carriers

Although this is an underpowered assessment of double mutations with only 23 patients, we thought that a descriptive and exploratory analysis is still warranted for pathogenic mutation carriers in this cohort. Out of the 23 patients having a known genetic cause of PD, 7 patients also have an additional rare variant in either *DNAH1, STAB1, ANK2, SH3GL2*, and/or *NOD2* (Table 1). Two patients (L-295 and L-2501) with a *LRRK2* p.R1441C mutation also carry a *DNAH1* p.S1089G variant. Two sisters with the *STAB1* p.S1089G variant in our discovery cohort harbor a homozygous *PINK1* p.Q456X variant. One patient with a negative FH of PD and an AAO of 35 years is compound heterozygous for pathogenic *PRKN* variants (deletion of 3–4 and duplication of exon 7–12) and also an *ANK2* p.V3634D variant. Patient L-649 is a carrier of a heterozygous *PRKN* p.R275W and an *SH3GL2* p.G276V variant, with a positive FH of PD and an AAO of 16 years. Lastly, patient L-1888 had compound heterozygous *PRKN* variants (p.R275W and exon one duplication) and a *NOD2* p.G908R variant.

The MDSGene database was used as a comparison tool as a systematic approach to check if combination of mutations leads to a more severe phenotype. The MDSGene database aims to provide a comprehensive, systematic overview of published data on movement disorder patients and currently contains data on 1,613 different mutations, from 1,227 publications. To systematically assess this, we filtered for the pathogenic variants identified and the clinical features that are associated with the pathogenic variants. We found that the median AAO for a *LRRK2* p.R1441C heterozygous mutation carrier is at 58 years of age. In comparison, patient L-295 (with a *DNAH1* p.A3639T variant) had an AAO of 45 years. Patient L-649 with *SH3GL2* p.G276V and *PRKN* p.R275W has an earlier onset of 16 compared to the median AAO of 41 for *PRKN* p.R275W carriers. Lastly, the *NOD2* p.G908R variant carrier has an AAO of 19, which is earlier than the average *PRKN* compound heterozygous carrier of either the Ex1Dupl or *PRKN* p.R275W. The other double mutation carriers (patients with *STAB1* and *ANK2* variants) had AAO comparable to the median AAO of patients with mutations in a single gene (Supplementary Table 4).

### IPDGC Replication

We then further assessed the identified six variants that were present in two patients and no controls (*DNAH1* p.A3639T, *STAB1* p.S1089G, *ANK2* p.V3634D, *ANK2* p.R3906W, *SH3GL2* p.G276V, and *NOD2* p.G908R) in the IPDGC cohort. Significant association testing was found for three variants (*p* < 0.0001) with an odds ratio (OR) of 2.08 [95% confidence interval (95% CI), 1.40–3.08] for *SH3GL2* p.G276V, 3.45 (95% CI, 2.77–4.28) for *NOD2* p.G908R, and 2.87 (95% CI, 1.51–5.13) for *STAB1* p.S1089G (Supplementary Table 5).

## Discussion

Although GWAS associations in larger cohorts are valuable, there has not been a detailed evaluation of rare exome-derived variants. To investigate whether genes showing common variant association with PD in previously published GWAS loci also harbor rare variants of potential functional effect, we used exome sequencing in a German cohort and subsequent replication in the IPDGC exome data from patients of European descent. The power of our initial discovery cohort is a limitation of this study, as we only had 62 idiopathic PD patients. Considering the small sample size and lack of power, we attempted to replicate our findings in a larger cohort [IPDGC cohort (*n* = 2,859)]. Notably, previous studies have also yielded interesting candidates even with small sample sizes: Guo et al. have nominated interesting variants in *NUS1* from 39 trios with healthy parents and early-onset patients with PD (14), and a smaller study of exome sequencing in 50 early-onset patients with PD has nominated interesting variants in *SPG7* (11). The *SPG7* implication in PD has been independently identified (15). Taken together, although there are limitations, smaller discovery cohorts can still be valuable, and further replication in larger cohorts are warranted.

In this study, we used a CADD score of 15 as cutoff (e.g., top 5% most likely deleterious) to select rare variants which scored high for the deleteriousness. The >15 inclusion threshold has been suggested by the developers of CADD to identify potentially pathogenic variants, as it is the median value for all possible canonical splice site changes and non-synonymous variants (16). However, the optimal threshold of CADD C-score to uncover causal variants may depend on several factors. In this study, the particular interest regarding the contribution that low frequency and rare variants can have an impact on PD.

Thus, these variants have a higher probability of damaging functional impact. In the German cohort, 6 out of 54 variants were found in two or more patients and were not observed in healthy controls: *DNAH1* p.A3639T, *STAB1* p.S1089G, *ANK2* p.V3634D, *ANK2* p.R3906W, *SH3GL2* p.G276V, and *NOD2* p.G908R. Subsequently, in the IPDGC cohort, we detected a significant association with PD status (*p* < 0.001) for three variants: *STAB1* p.S1089G and *SH3GL2* p.G276V and *NOD2* p.G908R.

Unfortunately, no additional family members were available from patients with idiopathic PD to test for cosegregation with disease. However, evidence of association within a larger replication cohort suggests the importance of these low-frequency variants for PD risk. The OR is relatively low compared to usual OR for rare variants that are disease causing (range OR, 2.08–3.45); however, it is comparable to the *LRRK2* p.G2385R effect size. Thus, they seem to be weaker risk variants that might have an impact if present in a cumulative fashion within the same gene.

Although, we only have 23 patients with previously described pathogenic or likely pathogenic variants, we also identified several variants of interest that is worth further analysis. Since, we identified 6 variants of potential interest in these 71 genes in 85 PD cases, we used the probability of 6/85 (0.0697) as a reference. Thus, within 23 patients already carrying a pathogenic mutation, we would theoretically expect two patients with double mutations. Here, we identify seven patients with double mutations, which is more than the expected.

We utilized MDSGene (1, 2) database for further analysis and systematic comparisons across double mutation carriers for assessment of AAO. For example, L-649 had one *SH3GL2* variant and one heterozygous *PRKN* variant with an early AAO (16 years). *PRKN* heterozygous mutation carriers normally do not present with PD. However, patient L-649 with an *SH3GL2* variant had an even earlier onset of PD (AAO = 16) compared to the average *PRKN* homozygous p.R275W carrier (median AAO = 41). The average age of onset of all *PRKN* mutation carriers is 31 years (interquartile range, 23–38 years) (usually early onset patients are described as <45 years) (1). These two cases could indicate a modifying effect for AAO but cannot be determined in this study, as this is still on an exploratory level. How these rare variants contribute to disease pathology is of importance, and future mechanistic studies are necessary to elucidate the disease biology of these nominated GWAS candidates. SH3GL2 encodes endophilin A1, and it disrupts in the synaptic vesicle endocytosis and maintains axonal terminal integrity (17–20). The SH3GL2 p.G276V variant is within the N-terminal amphipathic helix Bin/Amphiphysin/Rvs-homology (N-BAR) domain, responsible for insertion into the lipid membrane through the BAR domain (19). SH3GL2 is also responsible for recruitment of dynamin to clathrin-coated vesicles (20). Parkin interacts with NOD2 for regulation of stress and inflammation; Parkin knockdown in mouse dopaminergic neurons exhibited increased NOD2 expression and endoplasmic reticulum stress and cytokine release (21). NOD2 deficiency was protective against 6-OHDA-induced DA degeneration and neuronal death (22), suggesting that the identified NOD2 variants in our patients may be toxic gain of function. The average AAO within the cases of the exome dataset was mostly early onset [AAO, 35.9 years (range, 6–80)], which is significantly younger than the AAO of patients from published GWAS. The differences between the AAO and the published GWAS studies and the potential differences in genetic etiology are a caveat in our study. Further studies with more patients would be warranted. However, using the IPDGC replication cohort and an additive model, with 2,859 cases and 24,146 controls, disease prevalence of 1%, MAF of 1%, and significance level of *p* = 0.0001, the sample was adequately powered (90.1%) to detect an effect of relative risk 2.0. Our study has identified rare variants in *STAB1, NOD2*, and *SH3GL2* within newly described GWAS loci, which may contribute to PD risk. All variants are reported in the Human Gene Mutation Database. Additional follow up of these variants, assessing familial segregation with PD, and functional studies are required to elucidate their possible impact on disease pathophysiology.

## Data Availability Statement

The raw data available supporting the conclusions of this manuscript will be made available by the authors, without undue reservation, to any qualified researcher.

## Ethics Statement

The studies involving human participants were reviewed and approved by Research Ethics Board of the University of Luebeck. The patients/participants provided their written informed consent to participate in this study.

## Author Contributions

EG, SI, and JT: (1) Research project: A. Conception, B. Organization, C. Execution; (2) Statistical Analysis: A. Design, B. Execution; (3) Manuscript: A. Writing of the first draft. CV-G and CK: (1) Research project: B. Organization, C. Execution; (2) Statistical Analysis: Review and Critique; (3) Manuscript: Review and Critique. PS, MK, and NB: (1) Research project: C. Execution; (2) Statistical Analysis: Review and Critique; (3) Manuscript: Review and Critique.

### Conflict of Interest

NB has received speaker's honoraria from Grünenthal, UCB, Abbvie, and Teva. He was employed as a consultant for Censa Pharmaceuticals. CK serves as a medical advisor to Centogene for genetic testing reports in the fields of movement disorders and dementia, excluding Parkinson's disease. The remaining authors declare that the research was conducted in the absence of any commercial or financial relationships that could be construed as a potential conflict of interest.

## References

[1] Kasten M Hartmann C Hampf J Schaake S Westenberger A Vollstedt EJ. Genotype-phenotype relations for the Parkinson's disease genes Parkin, PINK1, DJ1: MDSGene systematic review. Mov Disord. (2018) 33:730–41. 10.1002/mds.27352 29644727

[2] Trinh J Zeldenrust FMJ Huang J Kasten M Schaake S Petkovic S. Genotype-phenotype relations for the Parkinson's disease genes SNCA, LRRK2, VPS35: MDSGene systematic review. Mov Disord. (2018) 33:1857–70. 10.1002/mds.27527 30357936

[3] Keller DL. Parkinson disease. Cleve Clin J Med. (2012) 79:242–3; author reply 243, 248. 10.3949/ccjm.79c:04006 22473721

[4] Nalls MA Pankratz N Lill CM Do CB Hernandez DG Saad M. Large-scale meta-analysis of genome-wide association data identifies six new risk loci for Parkinson's disease. Nat Genet. (2014) 46:989–93. 10.1038/ng.3043 25064009 PMC4146673

[5] Polymeropoulos MH Lavedan C Leroy E Ide SE Dehejia A Dutra A. Mutation in the alpha-synuclein gene identified in families with Parkinson's disease. Science. (1997) 276:2045–7. 10.1126/science.276.5321.2045 9197268

[6] Simon-Sanchez J Schulte C Bras JM Sharma M Gibbs JR Berg D. Genome-wide association study reveals genetic risk underlying Parkinson's disease. Nat Genet. (2009) 41:1308–12. 10.1038/ng.487 19915575 PMC2787725

[7] Hulihan MM Ishihara-Paul L Kachergus J Warren L Amouri R Elango R. LRRK2 Gly2019Ser penetrance in Arab-Berber patients from Tunisia: a case-control genetic study. Lancet Neurol. (2008) 7:591–4. 10.1016/S1474-4422(08)70116-9 18539535

[8] Healy DG Wood NW Schapira AH. Test for LRRK2 mutations in patients with Parkinson's disease. Pract Neurol. (2008) 8:381–5. 10.1136/jnnp.2008.162420 19015299

[9] Chang D Nalls MA Hallgrimsdottir IB Hunkapiller J van der Brug M Cai F. A meta-analysis of genome-wide association studies identifies 17 new Parkinson's disease risk loci. Nat Genet. (2017) 49:1511–6. 10.1038/ng.3955 28892059 PMC5812477

[10] Nalls MA Blauwendraat C Vallerga CL Heilbron K Bandres-Ciga S Chang D Expanding Parkinson's disease genetics: novel risk loci, genomic context, causal insights and heritable risk. bioRxiv. (2019)388165 10.1101/388165

[11] Trinh J Lohmann K Baumann H Balck A Borsche M Bruggemann N. Utility and implications of exome sequencing in early-onset Parkinson's disease. Mov Disord. (2019) 34:133–7. 10.1002/mds.27559 30537300 PMC8950081

[12] Trujillano D Oprea GE Schmitz Y Bertoli-Avella AM Abou Jamra R Rolfs A. A comprehensive global genotype-phenotype database for rare diseases. Mol Genet Genomic Med. (2017) 5:66–75. 10.1002/mgg3.262 28116331 PMC5241210

[13] Karczewski KJ Francioli LC Tiao G Cummings BB Alföldi J Wang Q Variation across 141,456 human exomes and genomes reveals the spectrum of loss-of-function intolerance across human protein-coding genes. bioRxiv. (2019) 531210 10.1101/531210

[14] Guo JF Zhang L Li K Mei JP Xue J Chen J. Coding mutations in NUS1 contribute to Parkinson's disease. Proc Natl Acad Sci USA. (2018) 115:11567–72. 10.1073/pnas.1809969115 30348779 PMC6233099

[15] De la Casa-Fages B Fernandez-Eulate G Gamez J Barahona-Hernando R Moris G Garcia-Barcina M Parkinsonism and spastic paraplegia type 7: expanding the spectrum of mitochondrial Parkinsonism. Mov Disord. (2019) 34:1547–61. 10.1002/mds.27812 31433872

[16] Rentzsch P Witten D Cooper GM Shendure J Kircher M. CADD: predicting the deleteriousness of variants throughout the human genome. Nucleic Acids Res. (2019) 47:D886–94. 10.1093/nar/gky1016 30371827 PMC6323892

[17] Matta S Van Kolen K da Cunha R van den Bogaart G Mandemakers W Miskiewicz K. LRRK2 controls an EndoA phosphorylation cycle in synaptic endocytosis. Neuron. (2012) 75:1008–21. 10.1016/j.neuron.2012.08.022 22998870

[18] Milosevic I Giovedi S Lou X Raimondi A Collesi C Shen H. Recruitment of endophilin to clathrin-coated pit necks is required for efficient vesicle uncoating after fission. Neuron. (2011) 72:587–601. 10.1016/j.neuron.2011.08.029 22099461 PMC3258500

[19] Pechstein A Gerth F Milosevic I Japel M Eichhorn-Grunig M Vorontsova O. Vesicle uncoating regulated by SH3-SH3 domain-mediated complex formation between endophilin and intersectin at synapses. EMBO Rep. (2015) 16:232–9. 10.15252/embr.201439260 25520322 PMC4328750

[20] Yim YI Sun T Wu LG Raimondi A De Camilli P Eisenberg E. Endocytosis and clathrin-uncoating defects at synapses of auxilin knockout mice. Proc Natl Acad Sci USA. (2010) 107:4412–7. 10.1073/pnas.1000738107 20160091 PMC2840126

[21] Singh K Han K Tilve S Wu K Geller HM Sack MN. Parkin targets NOD2 to regulate astrocyte endoplasmic reticulum stress and inflammation. Glia. (2018) 66:2427–37. 10.1002/glia.23482 30378174 PMC6275110

[22] Cheng L Chen L Wei X Wang Y Ren Z Zeng S. NOD2 promotes dopaminergic degeneration regulated by NADPH oxidase 2 in 6-hydroxydopamine model of Parkinson's disease. J Neuroinflammation. (2018) 15:243. 10.1186/s12974-018-1289-z 30157869 PMC6116377

