# Supplementary material for: The Role of Rare Coding Variants in Parkinson's Disease GWAS Loci
Source: Front Neurol. 2019 Dec 13;10:1284. doi: 10.3389/fneur.2019.01284 (PMC6923768; doi:10.3389/fneur.2019.01284)
Supplement: Supplementary file 1 [file Data_Sheet_1.pdf]

**Supplementary Appendix**


| **Table of contents** | **Content** | **Page number** |
|---|---|---|
| Supplementary table 1 | Carriers of pathogenic variants | 2-3 |
| Supplementary table 2 | List of GWAS loci investigated | 4-5 |
| Supplementary table 3 | List of variants identified in 71 nominated genes | 6-8 |
| Supplementary table 4 | Double mutation carriers and clinical course | 9 |
| Supplementary table 5 | Single variant association testing | 10 |
| Acknowledgments | IPDGC | 11-13 |

**Supplementary table 1. Carriers of pathogenic variants**

| Patient ID | Variant identified | AAO | Gender | Family history | Tremor | Bradykinesia | Postural instability | Rigidity | Cognitive decline | Ethnicity |
|---|---|---|---|---|---|---|---|---|---|---|
| Likely genetically solved patients with (likely) pathogenic variants in the biallelic state for recessive or heterozygous state for dominant disorders | | | | | | | | | | |
| L-8599 | *PRKN* het p.R275W<br>*PRKN* het Ex2-4del | 17 | M | n.a. | yes | yes | n.a. | no | yes, MCI | German |
| L-513 | *PRKN* het p.Gln34Argfs*5<br>*PRKN* het Ex2-3del | 32 | F | yes | yes | n.a. | yes | yes | n.a. | German |
| L-3296 | *PRKN* het p.Asn52Metfs*29<br>*PRKN* het Ex6-7Dupl | 12 | F | yes | n.a. | n.a. | n.a. | n.a. | n.a. | German |
| B-11 | *PRKN* het Ex7 Del.+ het c.1072delT | 64 | M | yes | n.a. | n.a. | n.a. | n.a | n.a. | Italian |
| L-3035 | *PRKN* het Ex3+4 Del + het Ex7-9 Dupl | 31 | M | no | yes | n.a. | n.a. | n.a. | n.a. | n.a. |
| L-3048 | *PRKN* het p.A275T + het Ex4 Del | 15 | M | yes | n.a. | n.a. | n.a. | n.a. | n.a. | German |
| L-5415 | *PRKN* het p.R275W + het p.C352R | >35 | F | n.a. | n.a. | n.a. | n.a. | n.a. | n.a. | German |
| L-649 | *PRKN* het p.R275W | 16 | M | yes | yes | yes | yes | yes | n.a. | German |
| L-1888 | *PRKN* het p.R275W and Ex1dupl | 19 | F | no | yes | n.a. | n.a. | n.a. | n.a. | German |
| L-3043 | *PRKN* het p.P37L and *SNCA* Dupl | 33 | F | n.a. | n.a. | n.a. | n.a. | n.a. | n.a. | German |
| L-1703 | *PINK1* homo p.V170G | 31 | F | n.a. | n.a. | n.a. | n.a. | n.a. | n.a. | German |
| L-2122 | *PINK1* homo p.Gln456* | 61 | F | yes | n.a. | n.a. | n.a. | n.a. | n.a. | German |
| L-2123 | *PINK1* homo p.Gln456* | 39 | M | yes | n.a. | n.a. | n.a. | n.a. | n.a. | German |
| L-2124 | *PINK1* homo p.Gln456* | 53 | F | yes | n.a. | n.a. | n.a. | n.a. | n.a. | German |
| L-2126 | *PINK1* homo p.Gln456* | 47 | F | yes | n.a. | n.a. | n.a. | n.a. | n.a. | German |

| | | | | | | | | | | |
|---|---|---|---|---|---|---|---|---|---|---|
| L-1355 | *PLA2G6* het p.A781T<br>*PLA2G6* het p.Y790* | 24 | F | no | n.a. | n.a. | n.a. | n.a. | n.a. | German |
| L-1706 | *GBA* het p.R502C (p.R463C) | 35 | F | yes | n.a. | yes | yes | yes | no | German |
| L-1927 | *GBA* het p.L483P (p.L444P) | 30 | M | no | yes | yes | yes | yes | yes, MCI | German |
| L-865 | *GBA* het p.F252I (p.F213I) | 35 | F | n.a. | n.a. | n.a. | n.a. | n.a. | no | German |
| L-3512 | *GBA* het p.N409S | 39 | M | no. | n.a. | n.a. | n.a. | n.a. | n.a. | German |
| L-295 | *LRRK2* het p.R1441C | 45 | F | yes | n.a. | n.a. | n.a. | n.a. | n.a. | German |
| L-2501 | *LRRK2* het p.R1441C | >32 | M | yes | n.a. | n.a. | n.a. | n.a. | n.a. | German |
| L-7774 | *LRRK2* het p.R1441S | 51 | M | no. | yes | yes | no | yes | no | German |

Legend: AAO: age at onset, het: heterozygous, homo: homozygous, Dupl: duplication, Del: deletion, n.a.: not available, F: female, M: male, MCI: mild cognitive impairment

**Supplementary table 2. List of GWAS loci investigated**

| Chr: Position | SNP | Gene |
|---|---|---|
| 1:161469054 | rs6658353 | *FCGR2A* |
| 1:171719769 | rs11578699 | *VAMP4* |
| 1:226916078 | rs4653767 | *ITPKB* |
| 2:102413116 | rs34043159 | *IL1R2* |
| 2:166133632 | rs353116 | *SCN3A* |
| 2:18147848 | rs76116224 | *KCNS3* |
| 2:96000943 | rs2042477 | *KCNIP3* |
| 3:122196892 | rs55961674 | *KPNA1* |
| 3:151108965 | rs11707416 | *MED12L* |
| 3:161077630 | rs1450522 | *SPTSSB* |
| 3:18277488 | rs4073221 | *SATB1* |
| 3:28705690 | rs6808178 | *LINC00693* |
| 3:48748989 | rs12497850 | *NCKIPSD, CDC71* |
| 3:52816840 | rs143918452 | *ALAS1, ITIH4, ITIH3, TLR9, DNAH1, BAP1, PHF7, STAB1* |
| 4:114360372 | rs78738012 | *ANK2, CAMK2D* |
| 4:170583157 | rs62333164 | *CLCN3* |
| 4:17968811 | rs34025766 | *LCORL* |
| 5:102365794 | rs26431 | *PAM* |
| 5:134199105 | rs11950533 | *C5orf24* |
| 5:60273923 | rs2694528 | *ELOVL7* |
| 6:112243291 | rs997368 | *FYN* |
| 6:133210361 | rs75859381 | *RPS12* |
| 6:27681215 | rs9468199 | *ZNF184* |
| 6:30108683 | rs9261484 | *TRIM40* |
| 6:72487762 | rs12528068 | *RIMS1* |

| | | |
|---|---|---|
| 7:66009851 | rs76949143 | *GS1-124K5.11* |
| 8:130901909 | rs2086641 | *FAM49B* |
| 8:11707174 | rs2740594 | *CTSB* |
| 8:22525980 | rs2280104 | *SORBS3, BIN3, C8orf58, PDLIM2* |
| 9:17579690 | rs13294100 | *SH3GL2* |
| 9:34046391 | rs6476434 | *UBAP2* |
| 10:104015279 | rs10748818 | *GBF1* |
| 10:15569598 | rs10906923 | *FAM171A1* |
| 11:10558777 | rs7938782 | *RNF141* |
| 12:46419086 | rs7134559 | *SCAF11* |
| 13:133065768 | rs11610045 | *FBRSL1* |
| 13:49927732 | rs9568188 | *CAB39L* |
| 13:97865021 | rs4771268 | *MBNL2* |
| 14:37989270 | rs12147950 | *MIPOL1* |
| 14:75373034 | rs3742785 | *RPS6KL1* |
| 14:88472612 | rs8005172 | *GALC* |
| 16:28944396 | rs2904880 | *CD19* |
| 16:50736656 | rs6500328 | *NOD2* |
| 16:19279464 | rs11343 | *COQ7* |
| 16:52599188 | rs4784227 | *TOX3* |
| 16:58587672 | rs200564078 | *CNOT1* |
| 17:40698158 | rs601999 | *ATP6V0A1, PSMC3IP, TUBG2* |
| 17:42294337 | rs2269906 | *UBTF* |
| 17:42434630 | rs850738 | *FAM171A2* |
| 17:59917366 | rs61169879 | *BRIP1* |
| 17:76425480 | rs666463 | *DNAH17* |
| 17:7355621 | rs12600861 | *CHRNB1* |
| 18:31304318 | rs1941685 | *ASXL3* |

| | | |
|---|---|---|
| 18:48683589 | rs8087969 | *MEX3C* |
| 20:6006041 | rs77351827 | *CRLS1* |

Chr: chromosome, Position: hg19, Gene: HUGO nomenclature

**Supplementary table 3. List of variants identified in 61 nominated genes**

| Gene | Chr | Position (hg19) | Ref | Alt | SNP | Amino acid change | ExAC | GnomAD | CADD score | Cases (n) | Controls (n) |
|---|---|---|---|---|---|---|---|---|---|---|---|
| *SCN3A* | 2 | 165984439 | C | G | rs147300771 | SCN3A:NM_001081676: exon18:c.2948G>C:p.R983T | $8.25x10^{-3}$ | $1.59x10^{-5}$ | 18.87 | 1 | |
| *SCN3A* | 2 | 166032720 | G | A | n.a. | SCN3A:NM_001081676: exon3:c.185C>T:p.P62L | n.a. | n.a. | 28.6 | | 1 |
| *ALAS1* | 3 | 52237963 | A | G | n.a. | ALAS1:NM_001304443: exon4:c.512A>G:p.Q171R | n.a. | n.a. | 15.3 | 1 | |
| *DNAH1* | 3 | 52404770 | G | T | rs375219203 | DNAH1:NM_015512: exon41:c.6454G>T:p.D2152Y | 0.0004 | 0.0002082 | 25.8 | 1 | |
| *DNAH1* | 3 | 52426643 | G | A | rs201752275 | DNAH1:NM_015512: exon64:c.10216G>A:p.V3406I | 0.0031 | 0.002509 | 18.1 | 1 | |
| *DNAH1* | 3 | 52380680 | G | C | n.a. | DNAH1:NM_015512: exon11:c.1849G>C:p.D617H | n.a. | n.a. | 16.7 | 1 | |
| *DNAH1* | 3 | 52396410 | C | T | rs17052097 | DNAH1:NM_015512: exon31:c.4987C>T:p.R1663C | 0.0068 | $3.57x10^{-6}$ | 20.8 | 1 | |
| *DNAH1* | 3 | 52429022 | G | A | rs138940904 | DNAH1:NM_015512: exon68:c.10915G>A:p.A3639T | 0.0016 | 0.001262 | 34 | 3 | |
| *DNAH1* | 3 | 52410004 | G | A | rs201299120 | DNAH1:NM_015512: exon46:c.7193G>A:p.R2398H | 0.0005 | 0.00203 | 23.9 | 1 | |
| *DNAH1* | 3 | 52384594 | A | G | rs61734644 | DNAH1:NM_015512 :exon16:c.2717A>G:p.D906G | 0.0008 | 0.004475 | 24.5 | 1 | |
| *DNAH1* | 3 | 52424975 | C | G | rs200158571 | DNAH1:NM_015512: exon61:c.9646C>G:p.L3216V | n.a. | 0.0006666 | 23.5 | | 1 |
| *DNAH1* | 3 | 52398697 | C | T | rs61739896 | DNAH1:NM_015512: exon33:c.5288C>T:p.S1763L | 0.0679 | 0.006287 | 27.8 | 1 | |
| *DNAH1* | 3 | 52422300 | C | T | rs61731638 | DNAH1:NM_015512: exon57:c.9121C>T:p.R3041C | 0.0811 | 0.006178 | 35 | 1 | |
| *DNAH1* | 3 | 52400807 | G | T | rs200859252 | DNAH1:NM_015512: exon36:c.5669G>T:p.G1890V | n.a. | 0.002275 | 27 | 1 | |
| *DNAH1* | 3 | 52378570 | A | G | rs76591348 | DNAH1:NM_015512: exon9:c.1351A>G:p.K451E | 0.0005 | 0.0009621 | 20.4 | 1 | |

| | | | | | | | | | | | |
|---|---|---|---|---|---|---|---|---|---|---|---|
| *ITIH3* | 3 | 52836533 | G | A | rs74320783 | ITIH3:NM_002217: exon12:c.1567G>A:p.G523R | 0.0087 | 0.007854 | 33 | 1 | |
| *ITIH3* | 3 | 52833078 | G | A | rs199634029 | ITIH3:NM_002217: exon7:c.760G>A:p.V254M | 0.0003 | 0.0002686 | 31 | 1 | |
| *ITIH4* | 3 | 52857988 | C | T | rs74971919 | ITIH4:NM_001166449: exon10:c.1204G>A:p.V402M | 0.0013 | 0.001455 | 25 | 1 | |
| *NISCH* | 3 | 52522297 | G | A | rs150644559 | NISCH:NM_007184: exon16:c.2789G>A:p.R930H | 0.007 | 0.008041 | 17.21 | 1 | |
| *SATB1* | 3 | 18458436 | G | T | n.a. | SATB1:NM_001131010: exon3:c.346C>A:p.L116M | n.a. | n.a. | 24.9 | 1 | |
| *STAB1* | 3 | 52548194 | C | T | rs139838594 | STAB1:NM_015136: exon33:c.3511C>T:p.R1171C | 0.0022 | 0.001995 | 16.71 | 1 | |
| *STAB1* | 3 | 52547252 | A | G | rs41292856 | STAB1:NM_015136: exon30:c.3265A>G:p.S1089G | 0.0003 | 0.001815 | 15.29 | 2 | |
| *TLR9* | 3 | 52255901 | C | T | rs201411213 | TLR9:NM_017442: exon2:c.2431G>A:p.D811N | $8.48x10^{-3}$ | $2.13x10^{-5}$ | 26.7 | 1 | |
| *ANK2* | 4 | 114279628 | T | C | rs36210417 | ANK2: NM_001148: exon38:c.9854T>C:p.I3285T | 0.0082 | 0.008634 | 25 | | 1 |
| *ANK2* | 4 | 114278578 | C | A | n.a. | ANK2:NM_001148: exon38:c.8804C>A:p.S2935Y | n.a. | n.a. | 19.71 | 1 | |
| *ANK2* | 4 | 114286207 | T | A | rs66785829 | ANK2: NM_001148: exon41: c.10901T>A: p.V3634D | 0.0027 | 0.002367 | 24.7 | 4 | |
| *ANK2* | 4 | 114254358 | G | C | n.a. | ANK2:NM_001148: exon29:c.3373G>C:p.D1125H | n.a. | $1.061x10^{-5}$ | 26.2 | 1 | |
| *ANK2* | 4 | 114279597 | G | A | n.a. | ANK2:NM_001148: exon38:c.9823G>A:p.D3275N | n.a. | $1.19x10^{-5}$ | 24.6 | 1 | |
| *ANK2* | 4 | 114275451 | G | T | n.a. | ANK2:NM_001148: exon38:c.5677G>T:p.V1893L | n.a. | n.a. | 22.5 | 1 | |
| *ANK2* | 4 | 114279549 | T | C | n.a. | ANK2:NM_001148: exon38:c.9775T>C:p.S3259P | n.a. | n.a. | 21.4 | | 1 |
| *ANK2* | 4 | 114294462 | C | T | rs121912706 | ANK2: NM_001148: exon43: c.11716C>T: p.R3906W | 0.0011 | 0.001066 | 20.9 | 2 | |
| *CLCN3* | 4 | 170641112 | G | A | rs149400550 | CLCN3:NM_173872: exon14:c.G2497G>A:p.G833S | 0.0043 | 0.004556 | 21.5 | 1 | |
| *PAM* | 5 | 102309844 | A | G | rs111855745 | PAM:NM_000919: exon14:c.1187A>G:p.K396R | 0.0001 | $7.98x10^{-5}$ | 20.9 | 1 | |
| *PAM* | 5 | 102282589 | C | T | rs78753846 | PAM:NM_000919: exon7:c.575C>T:p.P192L | 0.0081 | 0.00674 | 27.8 | 1 | |
| *RIMS1* | 6 | 72678706 | C | T | n.a. | RIMS1:NM_014989: exon2:c.185C>T:p.A62V | $3.31x10^{-2}$ | $2.00x10^{-5}$ | 17.15 | 1 | |
| *ZNF184* | 6 | 27420048 | G | C | rs61736956 | ZNF184:NM_007149: exon6:c.1290C>G:p.H430Q | 0.0097 | 0.009688 | 19.9 | 1 | |
| *C8orf58* | 8 | 22458457 | C | G | rs145988500 | C8orf58:NM_001013842: exon2:c.103C>G:p.R35G | 0.0095 | 0.009268 | 27 | 1 | |

| | | | | | | | | | | | |
|---|---|---|---|---|---|---|---|---|---|---|---|
| *SORBS3* | 8 | 22423972 | C | G | rs150705192 | SORBS3:NM_001018003: exon2:c.39C>G:p.D13E | 0.0036 | 0.003543 | 27 | 1 | |
| *SH3GL2* | 9 | 17793463 | G | T | rs150543523 | SH3GL2:NM_003026: exon8:c.G827T:p.G276V | 0.0035 | 0.003668 | 24.4 | 3 | |
| *UBAP2* | 9 | 33956096 | G | T | rs200331065 | UBAP2:NM_018449: exon11:c.847C>A:p.H283N | 0.0004 | 0.0003825 | 17.16 | 1 | |
| *FAM171A1* | 10 | 15296725 | C | T | n.a. | FAM171A1:NM_001010924: exon4:c.572G>A:p.G191E | n.a. | n.a. | 16.23 | 1 | |
| *FAM171A1* | 10 | 15262960 | A | C | rs780797236 | FAM171A1:NM_001010924: exon6:c.854T>G:p.M285R | $8.26X10^{-3}$ | $1.195x10^{-5}$ | 28.9 | 1 | |
| *RNF141* | 11 | 10546851 | T | C | rs61760882 | RNF141:NM_016422: exon4:c.322A>G:p.K108E | 0.0026 | 0.002802 | 18.21 | 1 | |
| *SCAF11* | 12 | 46320272 | C | T | rs146183261 | SCAF11:NM_004719: exon11:c.3212G>A:p.R1071H | 0.0001 | 7.077E-06 | 19.83 | 1 | |
| *SCAF11* | 12 | 46345447 | C | T | rs201119358 | SCAF11:NM_004719: exon4:c.283G>A:p.E95K | 0.0009 | 0.0008595 | 23.6 | 1 | |
| *NOD2* | 16 | 50750810 | A | G | rs104895467 | NOD2: NM_022162: exon6:c.2555A>G:p.N852S | 0.0012 | 0.001092 | 18.52 | 1 | |
| *NOD2* | 16 | 50745929 | C | T | rs5743277 | NOD2: NM_022162: exon4:c.2107C>T:p.R703C | 0.0033 | 0.003167 | 16.15 | 1 | |
| *NOD2* | 16 | 50756540 | G | C | rs2066845 | NOD2:NM_022162: exon8:c.2722G>C:p.G908R | 0.0099 | 0.0002015 | 23 | 2 | |
| *NOD2* | 16 | 50746086 | C | T | rs61747625 | NOD2: NM_022162: exon4:c.2264C>T:p.A755V | 0.0023 | 0.002519 | 24.4 | 1 | |
| *TOX3* | 16 | 52497869 | G | A | rs201752610 | TOX3:NM_001080430: exon3:c.385C>T:p.L129F | 0.0007 | 0.0006746 | 22.4 | | 1 |
| *FAM171A2* | 17 | 42433912 | G | T | rs150823411 | FAM171A2:NM_198475: exon4:c.484C>A:p.R162S | 0.0017 | 0.001046 | 22.2 | 1 | |
| *PSMC3IP* | 17 | 40729267 | C | A | rs139657728 | PSMC3IP:NM_013290: exon3:c.189G>T:p.K63N | 0.0005 | 0.0005303 | 26.6 | 1 | |
| *ASXL3* | 18 | 31326012 | T | G | rs144534810 | ASXL3:NM_030632: exon12:c.6200T>G:p.L2067R | 0.0063 | 0.006666 | 16 | 1 | |
| *CRLS1* | 20 | 5986989 | G | A | rs756560223 | CRLS1:NM_019095: exon1:c.97G>A:p.A33T | n.a. | 0.001086 | 15.2 | 1 | |

Legend: Chr: chromosome;Ref:reference allele; Alt: alternate allele; SNP: single nucleotide polymorphism; ExAC frequency: Exome Aggregation Consortium; GnomAD: Genome Aggregation Database CADD score: combined annotation dependent depletion score; Nr. of cases: numbers of cases; Nr. of controls: numbers of controls

**Supplementary table 4. Double mutation carriers and clinical course**

| MUTATION | ID | Sex | Age | AAO | FH | Population | Pathogenic variants | Median AAO of mutation carrier** | Initial signs/symptoms of mutation carrier** |
|---|---|---|---|---|---|---|---|---|---|
| *DNAH1 p.A3639T* | L-295 | female | n.a. | 45 | yes. | German | *LRRK2* het p.R1441C | 58 | Tremor (50% of patients) |
| | L-2501 | male | n.a. | n.a. | yes. | German | *LRRK2* het p.R1441C | 58 | Tremor (50% of patients) |
| *STAB1 p.S1089G* | L-2124 | female | 68 | 53 | yes | German | *PINK1* homo p.Gln456* | 37 | Bradykinesia (36% of patients) |
| | L-2126 | female | 72 | 47 | yes | German | *PINK1* homo p.Gln456* | 37 | Bradykinesia (36% of patients) |
| *ANK2 p.V3634D* | L-3035 | male | 40 | 35 | no | n.a. | *PRKN* het Ex3-4 Del.+ het Ex7-9 Dupl[%] | 34 | Tremor (50% of patients) |
| *SH3GL2 p.G276V* | L-649 | male | n.a. | 16 | yes | German | *PRKN* het p.R275W[$] | 41 | Tremor (50% of patients) |
| *NOD2 p.G908R* | L-1888 | female | 25 | 19 | no | German | *PRKN* het p.R275W and Ex1 Dupl[%] | 33 | Tremor (42% of patients) |

**Data obtained from MDSGene database, filtering for the specific pathogenic variants identified for summary clinical statistics. [$]=could not filter for heterozygous patients only as the database did not contain any heterozygous mutation carriers, thus filtered for homozgous. [%]filtered for any patient with compound heterozygous variants that include the specific pathogenic mutation of interest

**Supplementary table 5. Single variant association testing**

| Chr | Gene | Position (hg19) | Ref | Alt | Amino acid change | OR | 95% CI | p -value |
|---|---|---|---|---|---|---|---|---|
| 3 | *DNAH1* | 52429022 | G | A | p.A3639T | 1.20 | 0.52 to 2.76 | 0.08 |
| 3 | *STAB1* | 52547252 | A | G | p.S1089G | 2.87 | 1.61 to 5.13 | 0.0008 |
| 4 | *ANK2* | 114286207 | T | A | p.V3634D | 1.59 | 0.95 to 2.66 | 0.08 |
| 4 | *ANK2* | 114294462 | C | T | p.R3906W | 1.42 | 0.72 to 2.81 | 0.30 |
| 9 | *SH3GL2* | 17793463 | G | T | p.G276V | 2.08 | 1.40 to 3.08 | 0.0009 |
| 16 | *NOD2* | 50756540 | G | C | p.G908R | 3.45 | 2.77 to 4.28 | <0.0001 |

PD vs. healthy individuals are compared in this table. Legend: Chr: chromosome; Ref:reference allele; Alt: alternate allele; OR: odds ratio; CI: confidence interval

**IPDGC consortium members and affiliations:**

**United Kingdom:** Alastair J Noyce (Preventive Neurology Unit, Wolfson Institute of Preventive Medicine, QMUL, London, UK and Department of Molecular Neuroscience, UCL, London, UK), Arianna Tucci (Department of Molecular Neuroscience, UCL Institute of Neurology, London, UK), Demis A Kia (UCL Genetics Institute; and Department of Molecular Neuroscience, UCL Institute of Neurology, London, UK), Gavin Charlesworth (Department of Molecular Neuroscience, UCL Institute of Neurology, London, UK), Manuela Tan (Department of Clinical Neuroscience, University College London, London, UK), Henry Houlden (Department of Molecular Neuroscience, UCL Institute of Neurology, London, UK), Huw R Morris (Department of Clinical Neuroscience, University College London, London, UK), Helene Plun-Favreau (Department of Molecular Neuroscience, UCL Institute of Neurology, London, UK), Peter Holmans (Biostatistics & Bioinformatics Unit, Institute of Psychological Medicine and Clinical Neuroscience, MRC Centre for Neuropsychiatric Genetics & Genomics, Cardiff, UK), John Hardy (Department of Molecular Neuroscience, UCL Institute of Neurology, London, UK), Jose M Bras (Department of Molecular Neuroscience, UCL Institute of Neurology, London, UK), John Quinn (Institute of Translational Medicine, University of Liverpool, Liverpool, UK), Kin Y Mok (Department of Molecular Neuroscience, UCL Institute of Neurology, London, UK), Kimberley Billingsley (Institute of Translational Medicine, University of Liverpool, Liverpool, UK), Nicholas W Wood (UCL Genetics Institute; and Department of Molecular Neuroscience, UCL Institute of Neurology, London, UK), Patrick Lewis (University of Reading, Reading, UK), , Rita Guerreiro (Department of Molecular Neuroscience, UCL Institute of Neurology, London, UK), Ruth Lovering (University College London, London, UK), Raquel Duran Ogalla (University College London, London, UK), Lea R'Bibo (Department of Molecular Neuroscience, UCL Institute of Neurology, London, UK), Mina Ryten (Department of Molecular Neuroscience, UCL Institute of Neurology, London, UK), Valentina Escott-Price (MRC Centre for Neuropsychiatric Genetics and Genomics, Cardiff University School of Medicine, Cardiff, UK), Viorica Chelban (Department of Molecular Neuroscience, UCL Institute of Neurology, London, UK), Thomas Foltynie (UCL Institute of Neurology, London, UK), Una-Marie Sheerin (Department of Molecular Neuroscience, UCL Institute of Neurology, London, UK), Nigel Williams (MRC Centre for Neuropsychiatric Genetics and Genomics, Cardiff, UK),

**France:** Alexis Brice (Institut du Cerve au et de la Moelle épinière, ICM, Inserm U 1127, CNRS, UMR 7225, Sorbonne Universités, UPMC University Paris 06, UMR S 1127, AP-HP, Pitié-Salpêtrière Hospital, Paris, France), Fabrice Danjou (Institut du Cerveau et de la Moelle épinière, ICM, Inserm U 1127, CNRS, UMR 7225, Sorbonne Universités, UPMC University Paris 06, UMR S 1127, AP-HP, Pitié-Salpêtrière Hospital, Paris, France), Suzanne Lesage (Institut du Cerveau et de la Moelle épinière, ICM, Inserm U 1127, CNRS, UMR 7225, Sorbonne Universités, UPMC University Paris 06, UMR S 1127, AP-HP, Pitié-Salpêtrière Hospital, Paris, France), Jean-Christophe Corvol (Institut du Cerveau et de la Moelle épinière, ICM, Inserm U 1127, CNRS, UMR 7225, Sorbonne Universités, UPMC University Paris 06, UMR S 1127, Centre d'Investigation Clinique Pitié Neurosciences CIC-1422, AP-HP, Pitié-Salpêtrière Hospital, Paris, France), Maria Martinez (INSERM UMR 1220; and Paul Sabatier University, Toulouse, France),

**Germany:** Anamika Giri (Department for Neurodegenerative Diseases, Hertie Institute for Clinical Brain Research, University of Tübingen, and DZNE, German Center for Neurodegenerative Diseases, Tübingen, Germany), Angelika Oehmig (Department for Neurodegenerative Diseases, Hertie Institute for Clinical Brain Research, University of Tübingen, and DZNE, German Center for Neurodegenerative Diseases, Tübingen, Germany), Claudia Schulte (Department for Neurodegenerative Diseases, Hertie Institute for Clinical Brain Research), Kathrin Brockmann (Department for Neurodegenerative Diseases, Hertie Institute for Clinical Brain Research, University of Tübingen, and DZNE, German Center for Neurodegenerative Diseases, Tübingen, Germany), Javier Simón-Sánchez (Department for Neurodegenerative Diseases, Hertie Institute for Clinical Brain Research, University of Tübingen, and DZNE, German Center for Neurodegenerative Diseases, Tübingen, Germany), Peter Heutink (DZNE, German Center for Neurodegenerative Diseases and Department for Neurodegenerative Diseases, Hertie Institute for Clinical Brain Research, University of Tübingen, Tübingen, Germany), Patrizia Rizzu (DZNE, German Center for Neurodegenerative Diseases), Manu Sharma (Centre for Genetic Epidemiology, Institute for Clinical Epidemiology and Applied Biometry, University of Tubingen and Department for Neurodegenerative

Diseases, Hertie Institute for Clinical Brain Research, University of Tübingen Germany), Thomas Gasser (Department for Neurodegenerative Diseases, Hertie Institute for Clinical Brain Research, and DZNE, German Center for Neurodegenerative Diseases, Tübingen, Germany),

**United States of America:** Aude Nicolas (Laboratory of Neurogenetics, National Institute on Aging, Bethesda, MD, USA), Mark R Cookson (Laboratory of Neurogenetics, National Institute on Aging, Bethesda, USA), Sara Bandres-Ciga (Laboratory of Neurogenetics, National Institute on Aging, Bethesda, MD, USA), Cornelis Blauwendraat (National Institute on Aging and National Institute of Neurological Disorders and Stroke, USA), Faraz Faghri (Laboratory of Neurogenetics, National Institute on Aging, Bethesda, USA; Department of Computer Science, University of Illinois at Urbana-Champaign, Urbana, IL, USA), J Raphael Gibbs (Laboratory of Neurogenetics, National Institute on Aging, Bethesda, MD, USA), Dena G Hernandez (Laboratory of Neurogenetics, National Institute on Aging, Bethesda, MD, USA), Joshua M. Shulman (Baylor College of Medicine, Houston, Texas, USA), Mike A. Nalls (Laboratory of Neurogenetics, National Institute on Aging, Bethesda, USA; CEO/Consultant Data Tecnica International, Glen Echo, MD, USA), Laurie Robak (Baylor College of Medicine, Houston, Texas, USA), Steven Lubbe (Ken and Ruth Davee Department of Neurology, Northwestern University Feinberg School of Medicine, Chicago, IL, USA), Steven Finkbeiner (Departments of Neurology and Physiology, University of California, San Francisco; Gladstone Institute of Neurological Disease; Taube/Koret Center for Neurodegenerative Disease Research, San Francisco, CA, USA), Niccolo E. Mencacci (Northwestern University Feinberg School of Medicine, Chicago, IL, USA), Codrin Lungu (National Institutes of Health Division of Clinical Research, NINDS, National Institutes of Health, Bethesda, MD, USA), Andrew B Singleton (Laboratory of Neurogenetics, National Institute on Aging, Bethesda, MD, USA), Sonja Scholz (Neurodegenerative Diseases Research Unit, National Institute of Neurological Disorders and Stroke, Bethesda, MD, USA), Xylena Reed (Laboratory of Neurogenetics, National Institute on Aging, Bethesda, MD, USA).

**Canada:** Ziv Gan-Or (Montreal Neurological Institute and Hospital, Department of Neurology & Neurosurgery, Department of Human Genetics, McGill University, Montréal, QC, H3A 0G4, Canada), Guy A. Rouleau (Montreal Neurological Institute and Hospital, Department of Neurology & Neurosurgery, Department of Human Genetics, McGill University, Montréal, QC, H3A 0G4, Canada)

**The Netherlands:** Jacobus J van Hilten (Department of Neurology, Leiden University Medical Center, Leiden, Netherlands), Johan Marinus (Department of Neurology, Leiden University Medical Center, Leiden, Netherlands)

**Spain:** Juan A. Botía (Universidad de Murcia, Murcia, Spain), Jordi Clarimón (Memory Unit, Department of Neurology, IIB Sant Pau, Hospital de la Santa Creu i Sant Pau, Universitat Autònoma de Barcelona, Barcelona, and Centro de Investigación Biomédica en Red en Enfermedades Neurodegenerativas (CIBERNED), Madrid), Oriol Dols-Icardo (Memory Unit, Department of Neurology, IIB Sant Pau, Hospital de la Santa Creu i Sant Pau, Universitat Autònoma de Barcelona, Barcelona, and Centro de Investigación Biomédica en Red en Enfermedades Neurodegenerativas (CIBERNED), Madrid), Jaime Kulisevsky (Movement Disorders Unit, Department of Neurology, IIB Sant Pau, Hospital de la Santa Creu i Sant Pau, Universitat Autònoma de Barcelona, Barcelona, and Centro de Investigación Biomédica en Red en Enfermedades Neurodegenerativas (CIBERNED)), Javier Pagonabarraga (Movement Disorders Unit, Department of Neurology, IIB Sant Pau, Hospital de la Santa Creu i Sant Pau, Universitat Autònoma de Barcelona, Barcelona, and Centro de Investigación Biomédica en Red en Enfermedades Neurodegenerativas (CIBERNED)), Juan Marín (Movement Disorders Unit, Department of Neurology, IIB Sant Pau, Hospital de la Santa Creu i Sant Pau, Universitat Autònoma de Barcelona, Barcelona, and Centro de Investigación Biomédica en Red en Enfermedades Neurodegenerativas (CIBERNED)).

**Norway:** Lasse Pihlstrom (Department of Neurology, Oslo University Hospital, Oslo, Norway)

Estonia: Sulev Koks (Department of Pathophysiology, University of Tartu, Tartu, Estonia), Pille Taba (Department of Neurology and Neurosurgery, University of Tartu, Tartu, Estonia)
